# Inhibition of Nicotinic Acetylcholine Receptors, a Novel Facet in the Pleiotropic Activities of Snake Venom Phospholipases A_2_

**DOI:** 10.1371/journal.pone.0115428

**Published:** 2014-12-18

**Authors:** Catherine A. Vulfius, Igor E. Kasheverov, Vladislav G. Starkov, Alexey V. Osipov, Tatyana V. Andreeva, Sergey Yu. Filkin, Elena V. Gorbacheva, Maxim E. Astashev, Victor I. Tsetlin, Yuri N. Utkin

**Affiliations:** 1 Institute of Cell Biophysics, Russian Academy of Sciences, Ul. Institutskaya 3, Pushchino, Moscow Region 142290, Russia; 2 Shemyakin-Ovchinnikov Institute of Bioorganic Chemistry, Russian Academy of Sciences, Ul. Miklukho-Maklaya 16/10, Moscow 117997, Russia; Weizmann Institute of Science, Israel

## Abstract

Phospholipases A_2_ represent the most abundant family of snake venom proteins. They manifest an array of biological activities, which is constantly expanding. We have recently shown that a protein bitanarin, isolated from the venom of the puff adder *Bitis arietans* and possessing high phospholipolytic activity, interacts with different types of nicotinic acetylcholine receptors and with the acetylcholine-binding protein. To check if this property is characteristic to all venom phospholipases A_2_, we have studied the capability of these enzymes from other snakes to block the responses of *Lymnaea stagnalis* neurons to acetylcholine or cytisine and to inhibit α-bungarotoxin binding to nicotinic acetylcholine receptors and acetylcholine-binding proteins. Here we present the evidence that phospholipases A_2_ from venoms of vipers *Vipera ursinii* and *V. nikolskii*, cobra *Naja kaouthia*, and krait *Bungarus fasciatus* from different snake families suppress the acetylcholine- or cytisine-elicited currents in *L. stagnalis* neurons and compete with α-bungarotoxin for binding to muscle- and neuronal α7-types of nicotinic acetylcholine receptor, as well as to acetylcholine-binding proteins. As the phospholipase A_2_ content in venoms is quite high, under some conditions the activity found may contribute to the deleterious venom effects. The results obtained suggest that the ability to interact with nicotinic acetylcholine receptors may be a general property of snake venom phospholipases A_2_, which add a new target to the numerous activities of these enzymes.

## Introduction

Phospholipases A_2_ (PLA_2_s, phosphatidylcholine 2-acylhydrolase, EC 3.1.1.4) hydrolyze phospholipids and are usually most efficient on lipids with polyunsaturated fatty acids in the *sn*-2 position. They are classified into secretory, cytosolic, calcium-independent PLA_2_s as well as the platelet-activating factor acetylhydrolases, the lysosomal PLA_2_s, and adipose-specific PLA_2_s. In total there are 11 main groups of secretory PLA_2_s (designated by roman numerals) [1], [2] with further division into subgroups. PLA_2_s from snake venoms belong to groups IA, IB, IIA, and IIB. These enzymes constitute the largest family of snake venom proteins. Snake venom PLA_2_s of groups IA and IB are mainly monomers with molecular mass of 12–15 kDa excluding β-bungarotoxins which are heterodimers; the venoms of Elapidae snake family contain only PLA_2_s of these two groups. The venoms of Viperidae family contain only PLA_2_s of groups IIA and IIB. Some PLA_2_s of group II exist as dimers or higher oligomers composed of up to five subunits. PLA_2_s are major components of snake venom. For example, in Russell's viper *Vipera russelli* up to 70% of the protein content is PLA_2_s [3], present in the form of at least 7 isoenzymes. In all snake venoms PLA_2_s are represented by a large number of homologues and differ greatly in their toxicity and spectrum of biological activity. They manifest neurotoxicity, myotoxicity, cardiotoxicity, anticoagulant effect and some other pharmacological effects [4]. The array of PLA_2_ pharmacological effects is constantly expanding. For example, a PLA_2_ with thrombin-inhibiting activity was recently found [5].

Neurotoxic action of PLA_2_ appears as a blockade of neuromuscular transmission and usually follows several steps: a brief initial phase of weak inhibition of acetylcholine (ACh) release, a second prolonged phase of facilitated release, and a third phase of progressive decline of neurotransmission [6]. However, other neurotoxic mechanisms are also possible. We have recently found that bitanarin, a protein isolated from the venom of the puff adder *Bitis arietans*, has structural similarity to PLA_2_s from Viperidae snake venoms and possesses high Ca^2+^-dependent phospholipolytic activity. This protein was capable to block α7-similar nicotinic acetylcholine receptors (nAChRs) in *Lymnaea stagnalis* neurons and it competed with [^125^I]α-bungarotoxin (αBgt) for binding to human neuronal α7- and *Torpedo californica* ray muscle-type nAChRs, as well as to *L. stagnalis* acetylcholine-binding protein (AChBP) [7]. Basing on these data, we supposed that other snake venom PLA_2_s might also be active against nAChRs and recently reported in a short communication that some PLA_2_s suppressed ACh-elicited current in identified *L. stagnalis* neurons [8]. To examine this effect in more detail, we tested seven PLA_2_s from the venoms of *V. ursinii*, *V. nikolskii*, *Naja kaouthia*, and *Bungarus fasciatus* for their ability to diminish the currents evoked by agonists in the so-called identified giant *L. stagnalis* neurons which can be discerned from other neurons in the ganglia by their size (150–200 µ in diameter), bright color, specific position, and axon morphology. The neurons used in our study contain the nAChRs similar in pharmacological profile to neuronal α7 receptor type [9], [10], but are chloride rather than cationic ion channels [11]. However, the population of nAChRs is not homogenous there; at least two receptor types differing in the affinity for ACh and α-conotoxin ImI as well as in desensitization kinetics can be distinguished. The contribution of two types varies from one cell to another, time course of the response to ACh and current suppression by antagonists being dependent on the relative involvement of two receptor types. The nAChR type (nAChR-Ls-1) with faster desensitization, lower affinity for ACh and higher for α-conotoxin ImI is functionally closer to vertebrate α7 nAChR. To get more precise data about the action of PLA_2_s on just these receptors, we used either ACh or cytisine (Cyt) as the agonist after estimating the affinities of the receptors in the neuron under study. Cyt is known to be a more selective agonist for α7 and some heteromeric types of nAChR as compared to ACh. It is a very weak partial agonist for β2- or α6-containing nAChRs and completely inactive at α9 receptors; at the same time it is a full agonist for α7 and β4-containig nAChRs [12]–[16]. In *L. stagnalis* neurons, Cyt activates nAChR-Ls-1 with high affinity for α-conotoxin ImI and low for ACh, while nAChRs possessing low affinity for α-conotoxin ImI and high for ACh (nAChR-Ls-2) are insensitive to Cyt. Neurons containing both receptor types at different ratios were used in this study. In addition, bitanarin was reexamined on neurons containing nAChR-Ls-1. Moreover, we have tested some PLA_2_s in competition experiments with αBgt for binding to nAChRs and AChBPs. The results obtained indicate that the ability to interact with nAChRs may be the general property of snake venom PLA_2_s.

## Materials and Methods

### Materials

Trizma-HCl, EGTA, HEPES, Pronase E, acetylcholine iodide, Cyt, and all chloride salts were purchased from Sigma (USA). Mono-iodinated (3-[^125^I]iodotyrosyl^54^)-αBgt (∼2000 Ci/mmol) was from GE Healthcare. nAChR-enriched membranes from the electric organs of *T. californica* ray were kindly provided by Prof. F. Hucho (Free University of Berlin, Germany), GH_4_C_1_ cells transfected with human α7 nAChR were a gift from Eli-Lilly (USA). The expressed acetylcholine binding proteins (AChBP) from *L. stagnalis* and *Aplysia californica* were kindly provided by Prof. T. Sixma (Netherlands Cancer Institute, Amsterdam, the Netherlands). Venom samples from vipers *V. nikolskii* and *V. ursinii* were obtained in 2009 by milking the snakes kept in captivity at Tula zoo (the Tula Exotarium). The permission was issued by the zoo director S.A.Ryabov. The venom of krait *B. fasciatus* was collected from snakes obtained from the authorized, licensed local establishment for snake breeding and venom production with permission of its owner Mr. Ha Văn Ti
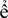
n (Vinh Sh
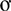
n, Vinh Tu'
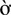
ng, Vinh Phúc province, Vietnam). Cobra (*N. kaouthia*) venom was obtained as described earlier [17]. All efforts were undertaken to minimize suffering of the animals while venom samples were collected. Venoms collected were dried over anhydrous CaCl_2_ and stored at −20°C until use.

### PLA_2_

**Figure 1:**
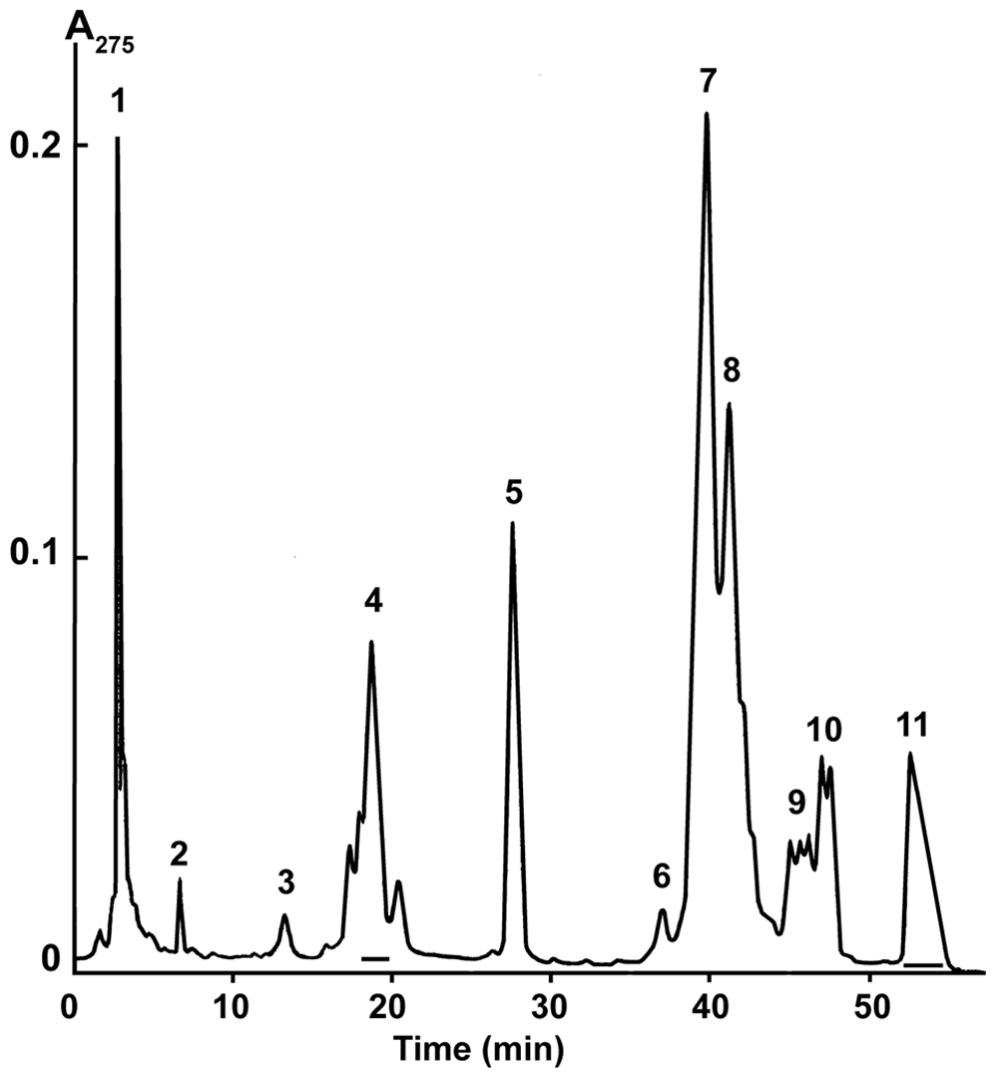
Isolation of vurtoxin and Vur-S49 by reverse-phase HPLC. Separation was done on a Discovery BIO Wide Pore C18 column (10×250 mm, Supelco) in a gradient of 25–40% (v/v) acetonitrile in 60 min in the presence of 0.1% (v/v) trifluoroacetic acid, at a flow rate of 2.0 ml/min. Fraction containing Vur-S49 (4) and vurtoxin (11) are indicated by horizontal bars.

PLA_2_ CM-II was purified from *N. kaouthia* venom as described [18]; PLA_2_ Vur-PL2 was isolated from *V. ursinii* venom as described [19]. The purity of the proteins was proved by analytical HPLC and mass spectrometry. PLA_2_s vurtoxin and Vur-S49 were isolated from *V. ursinii* venom. For this purpose, crude venom was separated by gel-filtration as described [19]. Fraction 3+4 (Figure 3A in [19]) was further purified by reversed phase HPLC on a Discovery BIO Wide Pore C18 column (10×250 mm, Supelco) in a gradient of 25–40% (v/v) acetonitrile in 60 min in the presence of 0.1% (v/v) trifluoroacetic acid, at a flow rate of 2.0 ml/min (Fig. 1). The protein from the fraction 11 possessed the molecular mass of 13868 Da (that is similar to the calculated mass of vurtoxin [19]), and was subjected to peptide mass fingerprinting as described [20]. The mass spectrometry data showed that the masses of the peptides obtained after trypsinolysis corresponded to those derived from vurtoxin, therefore the isolated protein is vurtoxin. The similar analysis of the protein from fraction 4 possessing the molecular mass of 13935 Da indicated that it is Vur-S49 [19]. PLA_2_ KBf VI from *B. fasciatus* venom was isolated by combination of gel-filtration, ion-exchange and reversed phase liquid chromatography. Peptide mass fingerprinting was used to confirm the structure of this protein. Bitanarin was isolated from *B. arietans* venom as described [7], and heterodimeric PLA2s HDP-1 and HDP-2 - from *V. nikolskii* venom [20].

### PLA_2_ activity

The enzymatic activity of PLA_2_s was measured using a synthetic fluorescent substrate 1-palmitoyl-2-(10-pyrenyldecanoyl)-*sn*-glycero-3-phosphocholine (Molecular probes, the Netherlands) as described [21].

### Electrophysiology

The experiments were carried out on giant identified (see above) neurons (LP1,2,3, RPV2,3; for the map of *L. stagnalis* ganglia see [22]) isolated from the left and right parietal ganglia as described [10]. Neurons were internally perfused with the solution (in mM: CsCl 95, CaCl_2_ 0.3, EGTA 2, HEPES 10, pH 7.2) and voltage-clamped at −60 mV [23]. Constant flow of the external solution (in mM: NaCl 92, KCl 1.6, BaCl_2_ 2, MgCl_2_ 1.5, Trizma-HCl 4, pH 7.6; Ba^2+^ was used instead of Ca^2+^ to avoid phospholipolytic action of the snake venom PLA_2_s on the cell membrane) was maintained, except the time of application of the agonists or neuron incubation with antagonists. Experiments with Vur-S49 were done using normal external solution containing 88 mM NaCl and 4 mM CaCl_2_. Depending on the neuron type acetylcholine iodide (ACh) or Cyt were applied on the whole cell surface by 4 s pulses with intervals not less than 6 min. Agonist-induced currents were monitored and digitized with a patch-clamp amplifier A-M Systems (USA), the data acquisition was performed using Digidata1200 B interface and pClamp6 software (Axon Instruments Inc., USA). The preparation of PLA_2_ solutions from frozen aliquots and incubation of neurons were performed as described earlier for polypeptides from *B. arietans* venom [7]. Concentrations of the agonists were chosen as close to EC_50_ value as possible. The effects were estimated by changes in the peak current amplitude induced by ACh or Cyt after 5-min incubation with PLA_2_ as compared to the control responses before treatment and after prolonged washing the toxin out. The neuron treatment for 5 min was earlier shown to be sufficient for achieving the equilibrium in binding of different polypeptides to *Lymnaea* nAChRs. Results obtained with ACh on neurons containing predominantly nAChR-Ls-1 and with Cyt on neurons of both types did not differ significantly and were combined for evaluating concentration dependence of the effect if not mentioned otherwise. IC_50_ values were calculated using Sigma plot 11.0 software by the Hill plot analysis.

### Receptor binding studies

For competition binding assays, suspensions of nAChR-rich membranes from *T. californica* ray electric organ (1.25 nM αBgt binding sites) in 20 mM Tris-HCl buffer, pH 8.0, containing 1 mg/ml bovine serum albumin (BSA), human α7 nAChR transfected GH_4_C_1_ cells (0.4 nM α-Bgt binding sites) in 20 mM Tris-HCl buffer, pH 8.0, containing 1 mg/ml BSA, or solutions of heterologously expressed AChBPs from *L. stagnalis* and *A. californica* (2.4 and 150 nM in phosphate buffer saline containing 0.7 mg/ml BSA and 0.05% Tween 20 (PBS-T)) were incubated for 90 min with various amounts of the PLA_2_s, followed by an additional 5 min incubation with 0.1–0.2 nM [^125^I]αBgt. Nonspecific binding was determined by preliminary incubation of the preparations with 10 µM α-cobratoxin. The membrane and cell suspensions were applied to glass GF/C filters (Whatman, Kent, England) presoaked in 0.25% polyethylenimine, and the unbound radioactivity was removed from the filter by washing (3×3 ml) with 20 mM Tris-HCl buffer, pH 8.0, containing 0.1 mg/ml BSA. The AChBP solutions were applied to two layers of DE-81 filters presoaked in PBS-T buffer, and washed (3×3 ml) with PBS-T buffer.

The radioactivity bound was determined using Wizard 1470 Automatic Gamma Counter (Perkin Elmer). The binding results were analyzed using ORIGIN 7.5 (OriginLab Corporation, Northampton, MA, USA) fitting to a one-site dose-response curve by Equation: % response  = 100/{1+ ([PLA_2_]/IC_50_)*^n^*}, where IC_50_ is the concentration at which 50% of the binding sites are inhibited and *n* is the Hill coefficient.

### Molecular modeling

Vurtoxin structures were constructed with the program MODELER 9v7 using ammodytoxin A (PDB ID: 3G8G) or ammodytoxin C (PDB ID: 3G8H) as templates. The refined structure of full size *T. marmorata* nAChR (PDB ID: 2BG9) and HEX (version 6.12) program were used for docking experiments. Models of the extracellular nAChR domains were constructed as described [24].

## Results

### PLA_2_s and their enzymatic activity

**Figure 2:**
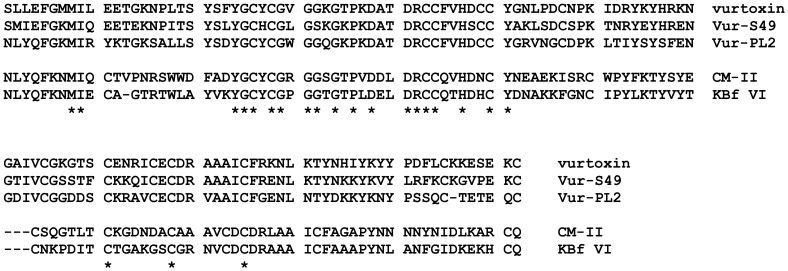
Amino acid sequences of 5 from 7 PLA_2_s used in the present study. CM-II (P00596) from *N. kaouthia*; KBf VI (P00627) from *B. fasciatus*; vurtoxin (F8QN54), Vur-S49 (F8QN50), and Vur-PL2 (F8QN53) from *V. ursinii*. Asterisks indicate identical residues.

In total seven different PLA_2_s from venoms of snakes belonging to the Elapidae and Viperidae families were analyzed, the amino acid sequences for five of them are shown in Fig. 2. CM-II and KBf VI PLA_2_s are from group IA, while vurtoxin, Vur-S49, Vur-PL2 as well as heterodimeric HDP-1 and HDP-2 from *V. nikolskii* are from group IIA. It was shown earlier that CM-II [18], KBf VI [25], Vur-PL2 [19], HDP-1, and HDP-2 [20] all possess strong enzymatic activity. Vur-S49 containing serine residue instead of aspartic acid at the position 49 of the active center represents enzymatically inactive PLA_2_ [26]. Testing of vurtoxin has shown that its activity is at a level typical for the snake PLA_2_s. The effect of Ba^2+^ ions in the Ca^2+^-free reaction mixture was examined on CM-II and it was found that in the presence of Ba^2+^ions CM-II becomes completely inactive. This effect is similar to that observed earlier for bitanarin [7]. The phospholipolytic activities of PLA_2_s studied are summarized in Table 1.

**Table 1:** Affinities for inhibiting different targets and phospholipolytic activities of PLA_2_s.

PLA_2_	Inhibition (IC_50_, µM) of α-[^125^I]Bgt Binding to…				Current Blocking and Phospholipolytic Activities	
	*T. californica* nAChR	Human α7 nAChR	*L. stagnalis* AChBP	*A. californica* AChBP	Inhibition (IC_50_, µM) of ACh (Cyt)-induced current in *L. stagnalis* neurons	Phospholipolytic activity, mmol/min/µmol
CM-II	1.2±0.1	3.2±0.3	1.00±0.03	>100	0.37±0.07*	2.3 [5]
Vurtoxin	0.26±0.02	14±5	>30	>30	10.5±2.6*	0.7
Vur-PL2	>100	29±2	>30	nd**	>30	25.1 [19]
Bitanarin	4.3±0.2 [7]	20±1.5 [7]	10.6±0.6 [7]	>50 [7]	4.8±1.3	1.95 [7]

* The data obtained for antagonizing action on currents induced by ACh in nAChR-Ls-1 neurons and by Cyt were combined

** Not determined

### Suppression of ACh – or Cyt - elicited currents by PLA_2_s from five snake species

**Figure 3:**
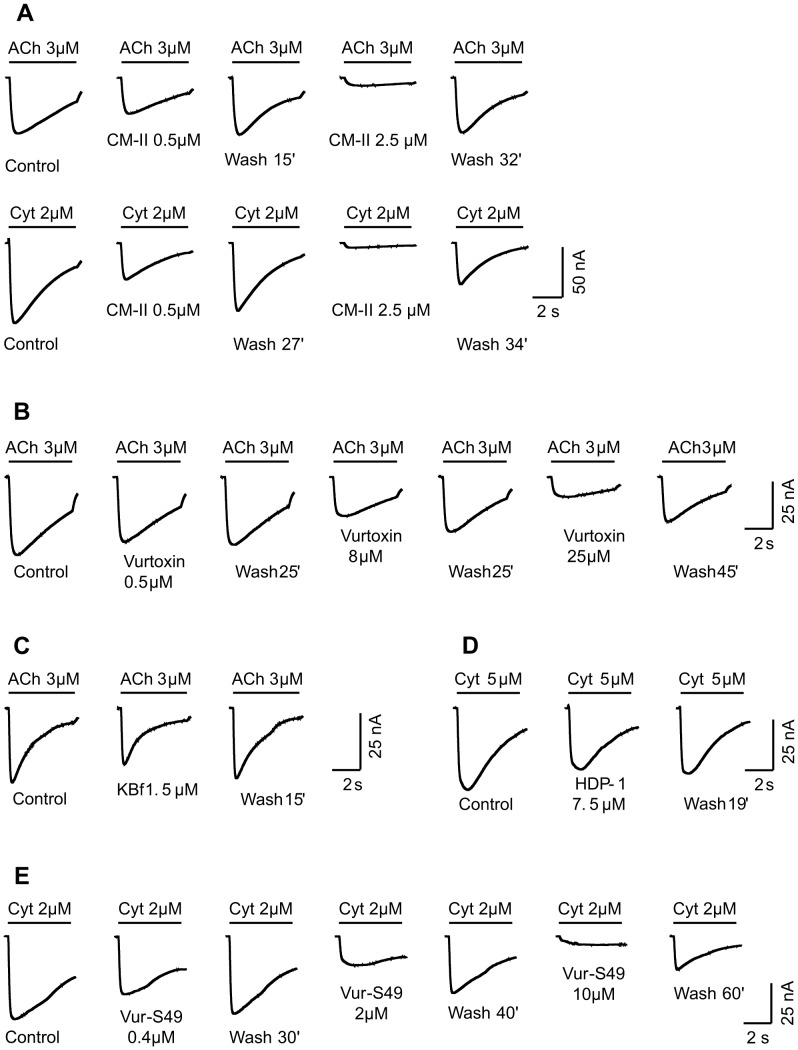
Inhibition of ACh- or Cyt-elicited current in *L. stagnalis* neurons by PLA_2_s from different snakes. *A* - CM-II from *N. kaouthia*; *B* - vurtoxin from *V. ursinii*; *C* – KBf VI from *B. fasciatus*; *D* – HDP-1 from *V. nikolskii*. *E* – Vur-S49 from *V. ursinii.* Duration of the treatment of the neurons with PLA_2_s was 5 min in all cases. The potency of CM-II as the antagonist was approximately the same while used against the ACh- or Cyt-induced current in nAChR-Ls-1 containing neuron (A). Very slow and incomplete recovery of the response to ACh (Cyt) is clearly seen after washing out of CM-II at 2.5 µM (A), vurtoxin at 8 and 25 µM (B) or Vur-S49 at 2 and 10 µM (E).

When tested on isolated *L. stagnalis* neurons, all seven PLA_2_s studied decreased the ACh- or Cyt-induced current. The results for five PLA_2_s are shown in Fig. 3A - E. PLA_2_ CM-II from *N. kaouthia* venom was the most potent. It decreased the current at concentration as low as 100 nM and almost complete block was observed at concentration of 2.5 µM (Fig. 3A). Vur-S49 was a slightly weaker antagonist (Fig. 3E) possessing IC_50_ of 2.18 µM. Other PLA_2_s inhibited the responses to ACh and Cyt in micromolar concentration range (Fig. 3B - D). The current recovered very slowly after washing PLA_2_s out, especially when the agent was used several times on a cell and/or at high concentration. After treatment with CM-II, bitanarin, Vur-S49 or vurtoxin at 2–10 µM, the response recovery was not complete even after 1 hour washing. In contrast, blockade by Vur-PL2 from *V. ursinii* venom was evident only at concentration of 10 µM or higher and was quickly reversible [8]. As the effects of PLA_2_s HDP-1, HDP-2 from *V. nikolskii* and KBf VI from *B. fasciatus* were similar to those described above, they were not analyzed in details. However, the inhibition of the ACh- or Cyt-elicited current by these PLA_2_s was clearly seen at concentration of 1.5–8 µM (Fig. 3C and D).

**Figure 4:**
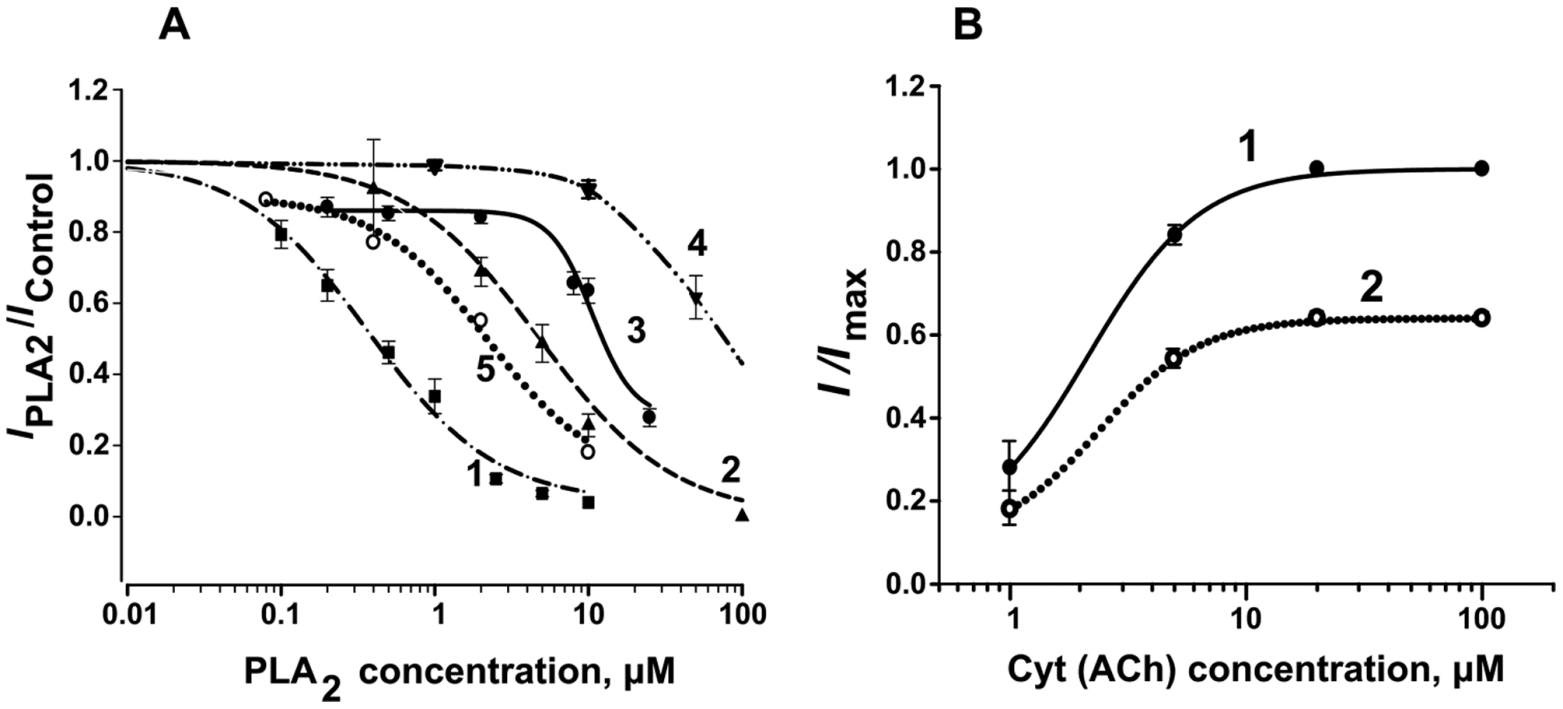
Concentration dependences of agonist-evoked currents in *L. staganalis* neurons. *A* – Dependence of agonist-evoked currents on concentrations of PLA_2_s. 1 (squares) - CM-II from *N. kaouthia* venom, 2 (triangles) - bitanarin from *B. arietans*, 3 (filled circles) – vurtoxin, 4 (reversed triangles) - Vur-PL2, 5 (open circles) - Vur-S49 from *V. ursinii*. IC_50_ values and Hill coefficients were for CM-II 0.37±0.07 µM and 1.06±0.18, for bitanarin 4.8±1.3 µM and 1.0±0.3, for vurtoxin 10.5±2.6 µM and 3.1±2.7, for Vur-PL2>30 µM, for Vur-S49 2.18±1.28 µM and 1.2±0.89. The data were obtained from 16, 7, 12, 6, and 9 cells, respectively. *B* –Dependence of the current elicited by either Cyt or ACh on agonist concentrations in control (1, filled circles) and after treatment with 2 µM Vur-S49 (2, open circles). EC_50_ and Hill slope values of 2.14 µM and 1.83, respectively, in control were changed to 2.38 µM and 2.00 after cell treatment with Vur-S49. The data were obtained from 3 neurons, one containing predominantly nAChR-Ls-1 and two with predominant nAChR-Ls-2, ACh being used on nAChR-Ls-1 neuron and Cyt on two nAChR-Ls-2 cells.

**Figure 5:**
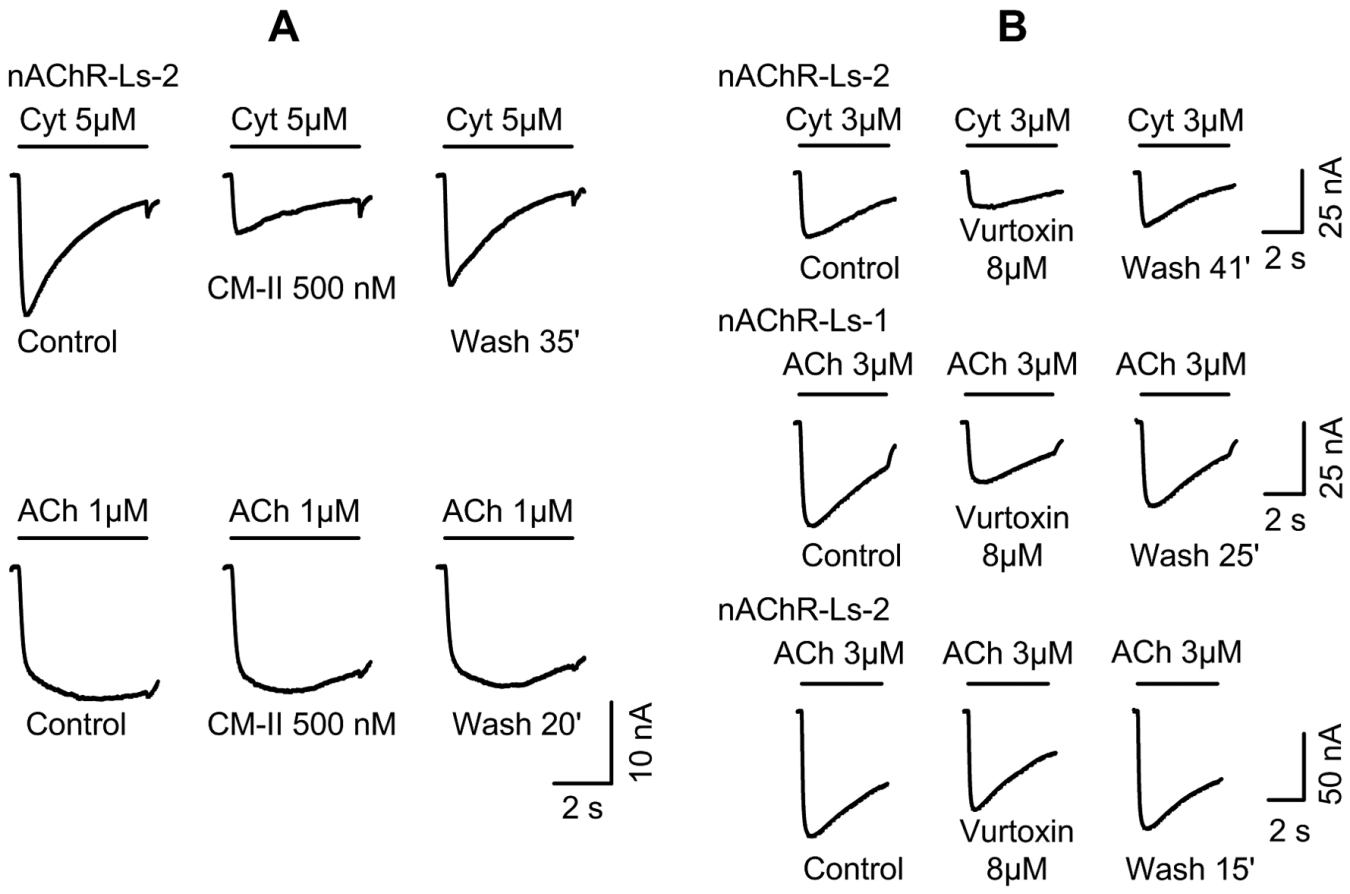
Comparison of CM-II and vurtoxin effects on Cyt- and ACh-evoked currents in neurons containing predominantly nAChR-Ls-2 or nAChR-Ls-1. *A* - A neuron with low sensitivity to ImI against ACh-induced current (nAChR-Ls-2). CM-II at concentration of 500 nM heavily decreased the Cyt-elicited current but affected very slightly the response to ACh. *B* - Vurtoxin at concentration of 8 µM decreased the currents elicited by Cyt or ACh in two neurons predominantly containing nAChR-Ls2 (up line) or nAChR-Ls1 (middle line) to 0.56 and 0.60 of the controls, respectively. In the third neuron with predominant nAChR-Ls-2 (bottom line) vurtoxin at the same concentration reduced the ACh-evoked current only to 0.80.

Concentration dependencies of the blockade of *L. stagnalis* neuron responses by PLA_2_s from *V. ursinii*, *N. kaouthia*, and *B. arietans* are shown in Fig. 4A. The calculated IC_50_ values are summarized in Table 1. When neurons containing predominantly nAChR-Ls-1 in the nAChR pool were chosen for experiments, blocking potency of bitanarin against ACh was 2.5 times higher than on neurons highly sensitive to ACh. The IC_50_ values for bitanarin were 4.8 µM on nAChR-Ls-1 and 11.4 µM on nAChR-Ls-2 [7]. The difference in IC_50_ values for CM-II determined for inhibition of the ACh-elicited current in two neuron types was even larger: for nAChR-Ls-2 neurons it was found to be 28.5 µM [8], whereas for nAChR-Ls-1 it was 0.37 µM. These data suggest that PLA_2_ capability to block agonist-elicited current in the neuron studied depends on the involvement of particular nAChR type in the interaction: with low affinity for α-conotoxin ImI and insensitive to Cyt or with high affinity for α-conotoxin ImI and activated by Cyt. This dependence is clearly seen when responses are elicited by ACh or Cyt in the same neuron containing predominantly nAChR-Ls-2 (Fig. 5A). CM-II at 500 nM strongly diminished the current induced by Cyt, but had practically no effect on the ACh-induced current. In other set of experiments, we compared the inhibition of the currents elicited by Cyt or ACh by vurtoxin in two types of neurons. As can be seen in Fig. 5 B, vurtoxin suppressed the responses to Cyt of nAChR-Ls-2 neuron (up line) and to ACh of nAChR-Ls-1 neuron (middle line) to the same extent. At the same time, the response to ACh of another nAChR-Ls-2 neuron (bottom line) was remarkably less sensitive to vurtoxin.

To determine if nAChR inhibition by PLA_2_s is competitive or noncompetitive, we analyzed the effect of Vur-S49 which possesses no phospholipolytic activity on the currents elicited by ACh or Cyt at different concentrations (including the saturating ones). The currents elicited by agonists were recorded before and after incubation with Vur-S49 at a constant concentration of 2 µM which is close to its IC_50_ value. One experiment with ACh on neuron containing predominantly nAChR-Ls-1 and two experiments with Cyt on neurons containing predominantly nAChR-Ls-2 were performed. As the relative inhibition of the responses to both agonists used at each concentration was very close we combined the data obtained in all three experiments. In the presence of Vur-S49, both the slope of the curve indicating the current dependence on agonist concentration and the maximum responses were clearly diminished (Fig. 4B). At the same time, EC_50_ and Hill coefficient values did not change significantly being 2.14 µM and 1.83 in control and 2.38 µM and 2.00 after neuron treatment with Vur-S49. These data suggest a noncompetitive type of the receptor inhibition.

### Analysis of PLA_2_s binding to nAChRs and AChBPs

**Figure 6:**
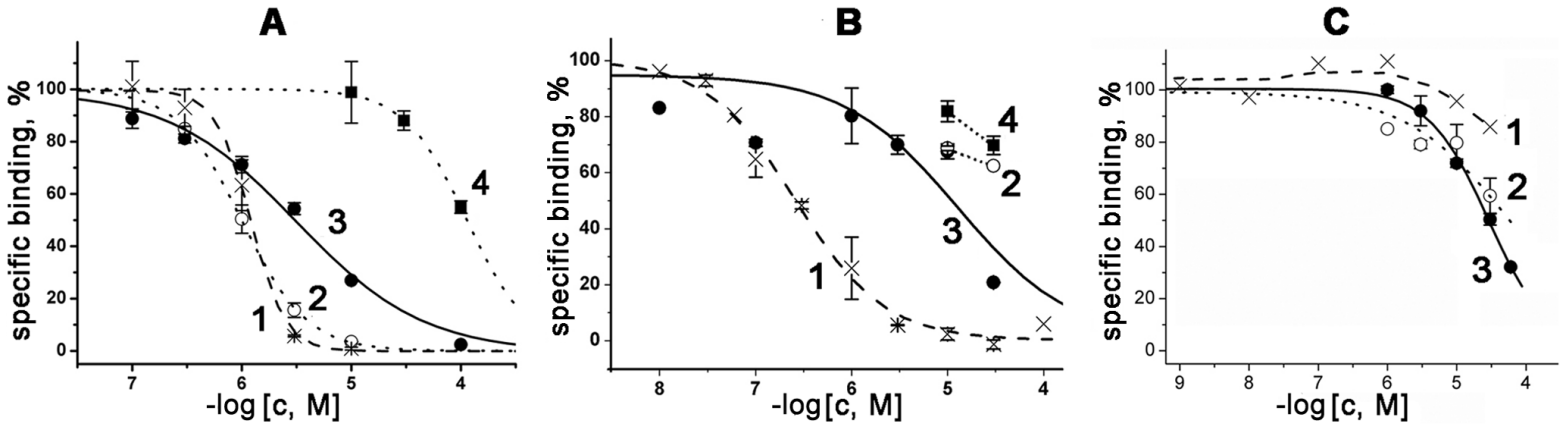
Radioligand analysis. Inhibition of [^125^I]-labeled α-Bgt binding by CM-II (*A*), vurtoxin (*B*) and Vur-PL2 (*C*) to *T. californica* (1, crosses, dashed line) and human α7 (3, filled circles, solid line) nAChRs as well as to *L. stagnalis* (2, open circles, dot line) and *A. californica* (4, filled squares, dot line) AChBPs. Each point is a mean ± s.e.m value of two or three measurements for each concentration. The curves were calculated from the means ± s.e.m. using ORIGIN 7.5 program. The calculated IC_50_ values are listed in Table 1.

For studying the capacity of PLA_2_s to bind to nAChRs, we used one neuronal- and one muscle-type nAChRs, as well as AChBPs from *L. stagnalis* and *A. californica* which differ in pharmacological profile. AChBPs, structural analogs of the extracellular ligand-binding domains for all nAChR subtypes, are widely used as models in receptor studies. The affinities of PLA_2_s for nAChRs and AChBPs were evaluated by radioligand assay in competition with [^125^I]-labeled αBgt as radioactive ligand. The membranes from electric organ of *T. californica* ray were used as a source of native muscle-type nAChR whereas transfected GH_4_C_1_ cells served as a source of human neuronal α7 nAChR [27]. Heterologously expressed soluble AChBPs from *L. stagnalis* and *A. californica* mollusks, which differ nearly 100-fold in potency of binding long-chain α-neurotoxins [28], [29] were used in these experiments. The data obtained showed that some studied PLA_2_s inhibited [^125^I]-labeled αBgt binding to tested targets, although with different potency. For example, *N. kaouthia* CM-II from the group IA interacted with close affinities with *T. californica* (IC_50_ 1.2 µM) and human α7 (3.2 µM) nAChRs as well as with *L. stagnalis* AChBP (1.0 µM), but practically did not bind *A. californica* AChBP (Fig. 6A). CM-II was found to be the most potent inhibitor of α7 nAChR among all tested PLA_2_s (see below) just as it was against native nAChRs in *L. stagnalis* neurons. On the other hand, vurtoxin was the most effective inhibitor of α-Bgt binding to *T. californica* nAChR with IC_50_ = 260±20 nM (Fig. 6B). Its interaction with α7 nAChR (IC_50_ = 14±5 µM,) and AChBPs (IC_50_>30 µM) was much less efficient. The IC_50_ value for vurtoxin interaction with α7 nAChR is very close to that on nAChR-Ls-1 in *L. staganalis* neurons. Vur-PL2 from the same *V. ursinii* venom and the same group IIA showed affinity for α7 nAChR (IC_50_ = 29±2 µM) similar to that of vurtoxin, but was practically inactive towards *T. californica* nAChR (Fig. 6C).

Although some tested PLA_2_s possess comparable phospholipolytic activities (Table 1), their apparent affinities for nAChRs differ greatly: 260 nM for vurtoxin versus>100 µM for Vur-PL2 at *T. californica* nAChR. The same is true for the affinities for AChBPs, which are water-soluble proteins. It should be mentioned that the PLA_2_ affinities for *A. californica* AChBP are much lower than for other targets. All calculated IC_50_ values are summarized in Table 1.

### Modeling of vurtoxin binding to nAChR

**Figure 7:**
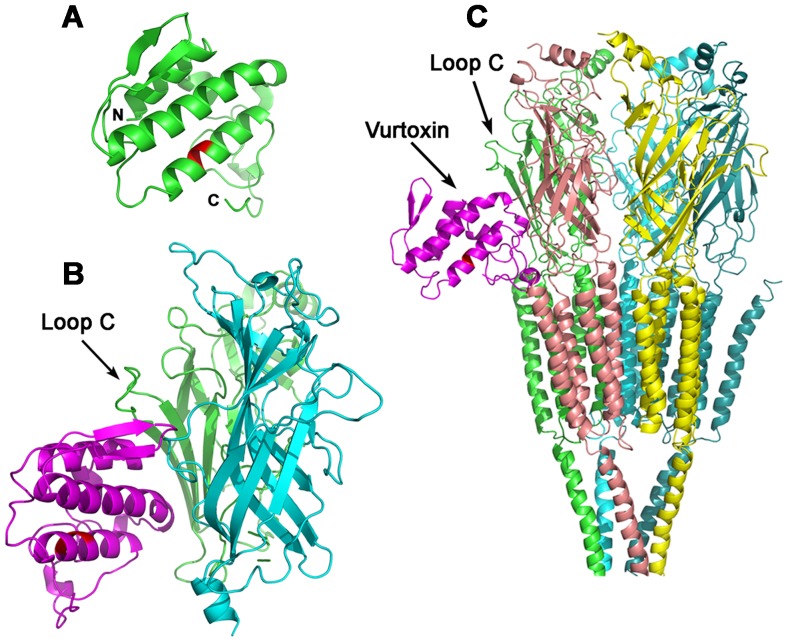
Molecular modelling of vurtoxin interaction with *Torpedo* nAChR. Vurtoxin active site residues are shown in red. *A* - Model of vurtoxin spatial structure. C and N indicate C- and N-termini, respectively. *B* - Model of vurtoxin complex with receptor extracellular domain at the α-γ subunit interface of the *T. californica* nAChR (vurtoxin is shown in magenta). *C* - Model of full size *Torpedo* nAChR complexed with vurtoxin at the α-γ subunit interface.

Since the amino acid sequence of vurtoxin has a very high degree of similarity to that of ammodytoxins [19], we constructed the model of vurtoxin spatial structure using the X-ray structures of ammodytoxins A and C as templates (Fig. 7A). The partial model of *T. californica* nAChR extracellular domain containing only α- and γ- subunits [24] and the whole *T. marmorata* receptor were used as targets. Docking experiments showed that vurtoxin is able to bind to both the extracellular domain and full size *Torpedo* nAChR (Fig. 7B, C). In both cases the binding was at the interface of subunits. Fig. 7B illustrates vurtoxin binding to the extracellular domain at α-γ subunit interface. Docking experiments with the full size receptor gave several solutions where vurtoxin is bound at the subunit interfaces close to the lipid bilayer. Vurtoxin was found to bind at α-β, α-δ and α-γ interfaces. PLA2 binding at α-γ interface was taken as an example. Fig. 7C shows docking solutions in which PLA_2_ molecule is docked at α-γ subunit interface, with the vurtoxin active site being oriented to the lipid bilayer. In this orientation vurtoxin molecule may be involved in binding to lipids in membrane surrounding the receptor. Vurtoxin does not occupy the classical binding site for agonists and competitive antagonists. However, its binding may prevent the movement of loop C (Fig. 7B) necessary for the activation of the receptor and sterically hinder the interaction of nAChR with α-Bgt.

## Discussion

Secretory PLA_2_s constitute a major component of snake venoms. These enzymes belong to the most toxic venom proteins and display a wide range of biological effects, neurotoxicity being one of them. The neurotoxicity of snake venom PLA_2_s is related to their action on presynaptic membrane of the synapse, therefore it is called presynaptic neurotoxicity. Some data suggest that this property is based on reduction of endocytosis that is strictly dependent on the phospholipolytic activity [30]. Nevertheless, a relation of neurotoxicity to catalytic activity of PLA_2_s still remains poorly understood and not always proved. For example, polyclonal antibodies to PLA_2_ from *V. russelli* venom did not affect PLA_2_ activity but neutralized neurotoxicity of PLA_2_s and venom as assayed by neurotoxic symptoms after i.p. injection into mice [31]. Chemical modification of several important amino acid residues or directed mutagenesis quite differently affected enzymatic activity and neurotoxic action of PLA_2_s from different snake venoms [32]–[34]. The N-terminal region was shown to be involved in neurotoxicity but not in catalytic activity of PLA_2_ OS2 from *Oxyuranus scutelletus* [33].

A hypothesis that high affinity binding of PLA_2_s to specific protein or glycoprotein targets is a primary prerequisite for toxicity was put forward by Kini and Evans [35]. This step might increase local concentration of PLA_2_ at some critical site of the membrane and accelerate phospholipolysis, otherwise it might promote pharmacological effects independent of enzymatic reaction [4], [35]–[37]. PLA_2_ binding proteins were purified from skeletal and smooth muscles, kidney, embryo fibroblasts, and cerebral cortex of mammals [38]–[41]. These proteins, named M- and N-receptors, had affinity for PLA_2_s in picomolar – nanomolar range. Recently it has been shown that the M-type receptor regulates cell senescence [42], promotes apoptosis and inhibition of proliferation and transformation thus manifesting tumor suppressive effects [43], and is involved in development of idiopathic membranous nephropathy [44].

Only few earlier reports mentioned a post-synaptic activity of snake venom PLA_2_s. Thus, it was shown that PLA_2_s from *B. caeruleus* and *Crotalus durissus terrificus* are able to stabilize the *Torpedo* nAChR in a desensitized state [45]–[47]. These PLA_2_s did not, however, interfere with the binding of *N. nigricollis* α-toxin to nAChR and could be considered noncompetitive antagonists. Moreover, it was shown that the treatment of *Torpedo* membranes with PLA_2_ did not alter the affinity of αBgt for nAChR [48].

In the present study we have shown that PLA_2_s from Viperidae and Elapidae snake venoms are capable to suppress the ACh (Cyt)-evoked current mediated by α7-similar nAChRs in *L. stagnalis* neurons and to compete with αBgt for binding to muscle- and α7 neuronal nAChR types, as well as to AChBPs. The apparent affinity of PLA_2_s for these targets varied from hundreds of nM to hundreds of µM depending on both the PLA_2_ studied and nAChR type (Table 1). The most active were vurtoxin against *T. californica* nAChR (0.26 µM) and CM-II against nAChRs in *L. stagnalis* neurons (0.37 µM). All other interactions were characterized by lower efficiencies with IC_50_ values spanning the range from micromoles to hundred micromoles (Table 1). It can be also seen that CM-II manifested no selectivity for muscle-type or neuronal α7 nAChRs but discriminated strongly AChBPs from the two mollusk species. In contrast, marked selectivity of vurtoxin was revealed: it was almost two orders of magnitude more active at *T. californica* nAChRs than at neuronal receptor type and AChBPs (see Table 1). In addition, differences in inhibitory activities when tested on two groups of *L. stagnalis* neurons, i.e. those with high and low sensitivity to ACh and inversed sensitivity to α-conotoxin ImI, should be emphasized. This difference was the highest (almost two orders of magnitude) for CM-II and smaller but significant (3–4 folds) for bitanarin and vurtoxin.

It is interesting to compare the potency of PLA_2_s to suppress ACh-induced current in *L. stagnalis* neurons containing predominantly nAChR-Ls-1 with that of neurotoxins from snake and snail venoms. Although IC_50_ values for PLA_2_s determined in this study are higher than those for α-cobratoxin (60 nM) or α-conotoxin ImI (10 nM) on the same neurons, they are of the same order as IC_50_s for a short α-neurotoxin NTII (11 µM) from *N. oxiana* and a weak neurotoxin WTX (67 µM) from *N. kaouthia* [9]. The potency of PLA_2_s in inhibiting ACh (Cyt)-induced current is comparable to that of αBgt in blocking the responses to ACh of *Aplysia* neurons (complete block was achieved at 1–10 µM, IC_50_ values varied from 50 nM to 1 µM) [49]. It should be mentioned that these values were several orders of magnitude higher than inhibition constant in experiments with ^125^I-αBgt binding to membrane preparations from Aplysia ganglia [49].

We have noted a slow reversibility of the current after washing out most of PLA2s. However, very slow and sometimes incomplete reversibility in the case of snake toxins acting on muscle-type and α7 nAChRs is a well known phenomenon. For example, binding of αBgt to α7 nAChR was not reversible even after 5 hours, after this time about 80% of toxin being bound to the receptor [50]. Other example includes αBgt binding to isolated rat skeletal-muscle fibers, containing muscle-type nAChRs; it has been characterized by extremely slow dissociation, a dissociation constant *k_d_* being less than or equal to 3×10^−6^ s^−1^ (half-life time more than 60 hours) [51]. Complete reversibility of the ACh-elicited current in *Aplysia* neuron after block by α-neurotoxin from *Nahja naja siamensis* venom required an hour and a half [49]. According to our earlier data [9], recovery of the ACh-induced current in *Lymnaea* neurons after treatment with α-cobratoxin also took more than one hour.

In some cases, incomplete recovery can be related also to the so-called rundown (i.e. falling down the response to a stimulus during several hour experiments). Rundown is an inevitable event which occurs even in the absence of any experimental influences as a result of a gradual decrease of excitable pool of channels under *in vitro* conditions. In our case, this process could be additionally accelerated by substitution of Ba^2+^ for Ca^2+^ in the extracellular medium. Thus, we considered recovery to 70–80% of the control values at the end of the experiment (in 2–3 hours after beginning) as acceptable value.

To get some insight into possible mechanism of PLA_2_ interaction with nAChRs, we have performed molecular modeling of vurtoxin binding to *Torpedo* receptor; the highest efficiency was observed for this interaction in binding assay. The molecular docking experiments were performed using the vurtoxin model constructed basing on the X-ray structures of ammodytoxins. Docking to the model of extracellular domain including only α- and γ-subunits resulted in vurtoxin binding at the interface of these subunits (Fig. 7B). In the model obtained, vurtoxin did not occupy the classical agonist binding site, but was located very close to it. When bound to the receptor, the toxin may hinder the movement of the loop C which is flexible and shows an inward displacement when certain agonists are attached. The existing data suggest that the loop C is involved in the receptor activation [52]. When docked to the whole receptor molecule, vurtoxin was also bound at the subunit interface (Fig. 7C). In this case, the binding site is located close to the membrane and one can suggest that in such position the PLA_2_ active center may interact with the membrane. In both models, vurtoxin does not block the classical ACh binding site completely; such position probably may explain comparatively low inhibitory activity of PLA_2_s. The modeling data are in good agreement with noncompetitive inhibition of agonist-elicited currents by Vur-S49 observed on *L. stagnalis* neurons (Fig. 4B).

Thus, our data show that PLA_2_s are capable to inhibit nAChRs and AChBPs. We believe that this inhibition is not the result of phospholipolytic activity. Several lines of evidence support our conclusion:

- Blockade was reversible (though slowly).
- There was no correlation between the capability to suppress the ACh- (Cyt-) elicited current and the catalytic activity of PLA_2_s. CM-II and bitanarin possess approximately equal catalytic activity (2.3 mmol/min/µmol and 1.95 mmol/min/µmol), but IC_50_ values for blocking the agonist-evoked current differ by an order of magnitude; Vur-PL2 is a very active PLA_2_ (specific activity 25.1 mmol/min/µmol) and the weakest current blocker or competitor of αBgt binding (see Table 1).
- Experiments on *L. stagnalis* neurons were conducted in Ca^2+^-free (Ba^2+^ substituted) solution. Ca^2+^ is known to be absolutely necessary for phospholipolytic activity of secretory PLA_2_s [53], [54]. In the absence of Ca^2+^ only traces of lipid hydrolysis products were found *in vitro* using bitanarin [7] and no hydrolysis at all was revealed with CM-II in the Ba^2+^-containing medium. Moreover, Ba^2+^ was shown to inhibit PLA_2_ activity [55].
- Perturbation of the cell membrane usually induces very quick morphological changes (turgor decrease well seen under microscope) and increase of electrical leakage. No such changes were observed under the action of PLA_2_s in the absence of Ca^2+^ in the extracellular medium.

Finally, the strongest evidences are the following:

- Vur-S49 possessing no phospholipolytic activity suppressed the agonist-evoked current and its inhibitory potency was higher than that of structurally similar but enzymatically active vurtoxin from the same venom (2.18 versus 10.5 µM).
- Comparison of CM-II effects on the responses of a neuron containing predominantly nAChR-Ls-2 (Fig. 5A) showed that CM-II at concentration 500 nM effectively decreased the current induced by Cyt but had hardly noticeable effect on the ACh-induced current in the same neuron. If the suppression was due to phospholipolytic activity the responses to both agonists should be diminished to approximately the same extent. A similar conclusion can be drawn from the data with vurtoxin effects on the currents in two types of neurons (Fig. 5B). The degree of reducing the ACh-evoked current in neurons containing predominantly nAChR-Ls-1 by vurtoxin didn't differ significantly from that of the Cyt-elicited current in both types of neurons, whereas in neurons of nAChR-Ls2 type vurtoxin was much weaker antagonist against the response to ACh. It could be hardly possible that phospholipids of nAChR-Ls-2 neuron were more resistant to enzymatic degradation.

The data obtained suggest that interaction of PLA_2_s with several types of nAChRs may be a general property. To our knowledge, binding of PLA_2_s to nAChRs was not yet described. Nevertheless, it was recently reported that PLA_2_ (P00602) from *N. mossambica* venom showed a low affinity AChBP binding [56]. Interaction of PLA_2_ with nAChRs could be involved in neurotoxic effect as an additional event. In particular, PLA_2_ binding to nAChRs in presynaptic membrane of nerve-muscle junction could abolish receptor modulating influence on ACh release.

In summary, we revealed antagonistic action of seven PLA_2_s from five snake species, three from Viperidae and two from Elapidae family, in electrophysiological experiments on nAChRs in native identified (LP1,2,3 and RPV2,3 [22]) neurons of *L. stagnalis* and in binding assay on two nAChR types as well as on two AChBPs. PLA_2_s suppressed the ACh- (Cyt)-elicited currents mediated by α7-similar nAChRs in *L. stagnalis* neurons and interfered with αBgt binding to human neuronal α7 and *T. californica* muscle-type nAChRs, as well as to AChBPs from *L. stagnalis* and *A. californica* mollusks. Thus, to an array of biological activities well-known for snake venom PLA_2_s, we have added the capability to block nAChRs.

## References

[1] Burke JE, Dennis EA (2009) Phospholipase A_2_ biochemistry. Cardiovasc Drugs Ther 23:49–59. 18931897 10.1007/s10557-008-6132-9 PMC2823292

[2] Schaloske RH, Dennis EA (2006) The phospholipase A_2_ superfamily and its group numbering system. Biochim Biophys Acta 1761:1246–1259. 16973413 10.1016/j.bbalip.2006.07.011

[3] Warrell DA (1989) Snake venoms in science and clinical medicine. 1. Russell's viper: biology, venom and treatment of bites. Trans Roy Soc Trop Med Hyg 3:732–740. 10.1016/0035-9203(89)90311-8 2533418

[4] Kini RM (2003) Excitement ahead: structure, function and mechanism of snake venom phospholipase A_2_ enzymes. Toxicon 42:827–840. 15019485 10.1016/j.toxicon.2003.11.002

[5] Osipov AV, Filkin SY, Makarova YV, Tsetlin VI, Utkin YN (2010) A new type of thrombin inhibitor, noncytotoxic phospholipase A_2_, from the Naja haje cobra venom. Toxicon 55:186–194. 19622365 10.1016/j.toxicon.2009.07.011

[6] Rossetto O, Morbiato L, Caccin P, Rigoni M, Montecucco C (2006) Presynaptic enzymatic neurotoxins. J Neurochem 97:1534–1545. 16805767 10.1111/j.1471-4159.2006.03965.x

[7] Vulfius CA, Gorbacheva EV, Starkov VG, Osipov AV, Kasheverov IE, et al (2011) An unusual phospholipase A_2_ from puff adder Bitis arietans venom – a novel blocker of nicotinic acetylcholine receptors. Toxicon 57:787–793. 21333664 10.1016/j.toxicon.2011.02.013

[8] Vulfius CA, Gorbacheva EV, Starkov VG, Kasheverov IE, Andreeva TV, et al (2013) Phospholipases A_2_ isolated from snake venoms block acetylcholine elicited currents in identified Lymnaea stagnalis neurons. Biochemistry (Moscow) Supplement Series A: Membrane and Cell Biology 7:203–206.

[9] Vulfius CA, Krasts IV, Utkin YN, Tsetlin VI (2001) Nicotinic receptors in Lymnaea stagnalis neurons are blocked by neurotoxins from cobra venoms. Neurosci Lett 309:189–192. 11514073 10.1016/s0304-3940(01)02081-x

[10] Vulfius CA, Tumina OB, Kasheverov IE, Utkin YN, Tsetlin VI (2005) Diversity of nicotinic receptors mediating Cl− current in Lymnaea neurons distinguished with specific agonists and antagonist. Neurosci Lett 373:232–236. 15619549 10.1016/j.neulet.2004.10.010

[11] Chemeris NK, Kazachenko VN, Kislov AN, Kurchikov AL (1982) Inhibition of acetylcholine responses by intracellular calcium in Lymnaea stagnalis neurons. J Physiol (London) 323:1–19. 6284913 10.1113/jphysiol.1982.sp014058 PMC1250342

[12] Papke RL, Heinemann SF (1994) The partial agonist properties of cytisine on neuronal nicotinic receptors containing β2 subunit. Mol Pharmacol 45:142–149. 8302273

[13] Buisson B, Gopalakrishnan M, Arneric SP, Sullivan JP, Bertrand D (1996) Human α4β2 neuronal nicotinic receptor in HEK 293 cells: A patch-clamp study. J Neurosci 16:7880–7891. 8987816 10.1523/JNEUROSCI.16-24-07880.1996 PMC6579202

[14] Gerzanich V, Kuryatov A, Anand R, Lindstrom J (1997) “Orphan” α6 nicotinic AChR subunit can form a functional heteromeric acetylcholine receptor. Mol Pharmacol 51:320–327. 9203638

[15] Wu J, Liu Q, Yu K, Hu J, Kuo YP, et al (2006) Roles of nicotinic acetylcholine receptor β subunits in function of human α4-containing nicotinic receptors. J Physiol (London) 576:103–118. 16825297 10.1113/jphysiol.2006.114645 PMC1995635

[16] Elgoyhen AB, Johnson DS, Boutler J, Vetter DE, Heinemann S (1994) α9: An acetylcholine receptor with novel pharmacological properties expressed in rat cochlear hair cells. Cell 79:705–715. 7954834 10.1016/0092-8674(94)90555-x

[17] Kukhtina VV, Weise C, Muranova TA, Starkov VG, Franke P, et al (2000) Muscarinic toxin-like proteins from cobra venom. Eur J Biochem 267:6784–6789. 11082188 10.1046/j.1432-1033.2000.01775.x

[18] Makarova YaV, Osipov AV, Tsetlin VI, Utkin YuN (2006) Influence of phospholipases A_2_ from snake venoms on survival and neurite outgrowth in pheochromocytoma cell line PC12. Biochemistry (Moscow) 71:838–846. 16827660 10.1134/s0006297906060125

[19] Tsai IH, Wang YM, Cheng AC, Starkov V, Osipov A, et al (2011) cDNA cloning, structural, and functional analyses of venom phospholipases A_2_ and a Kunitz-type protease inhibitor from steppe viper Vipera ursinii renardi. Toxicon 57:332–341. 21185324 10.1016/j.toxicon.2010.12.012

[20] Ramazanova AS, Zavada LL, Starkov VG, Kovyazina IV, Subbotina TF, et al (2008) Heterodimeric neurotoxic phospholipases A_2_ - The first proteins from venom of recently established species Vipera nikolskii: Implication of venom composition in viper systematics. Toxicon 51:524–537. 18083205 10.1016/j.toxicon.2007.11.001

[21] Radvanyi F, Jordan L, Russo-Marie F, Bon C (1989) A sensitive and continuous fluorometric assay for phospholipase A_2_ using pyrenelabeled phospholipids in the presence of serum albumin. Anal Biochem 177:103–109. 2742139 10.1016/0003-2697(89)90022-5

[22] Benjamin PR, Ings CT (1972) Golgi-Cox studies on the central nervous system of a gastropod mollusk. Z Zellforsch 128:564–582. 4112391 10.1007/BF00306989

[23] Kostyuk PG, Krishtal OA, Pidoplichko VI (1981) Intracellular perfusion. J Neurosci Methods 4:201–210. 6272031 10.1016/0165-0270(81)90032-7

[24] Mordvintsev DY, Polyak YL, Levtsova OV, Tourleigh YV, Kasheverov IE, et al (2005) A model for short alpha-neurotoxin bound to nicotinic acetylcholine receptor from Torpedo californica: comparison with long-chain alpha-neurotoxins and alpha-conotoxins. Comput Biol Chem 29:398–411. 16290328 10.1016/j.compbiolchem.2005.08.007

[25] Tsai IH, Tsai HY, Saha A, Gomes A (2007) Sequences, geographic variations and molecular phylogeny of venom phospholipases and threefinger toxins of eastern India Bungarus fasciatus and kinetic analyses of its Pro31 phospholipases A_2_ . FEBS J 274:512–525. 17166178 10.1111/j.1742-4658.2006.05598.x

[26] Rufini S, Cesaroni P, Desideri A, Farias R, Gubensek F, et al (1992) Calcium ion independent membrane leakage induced by phospholipase-like myotoxins. Biochemistry 31:12424–12430. 1334427 10.1021/bi00164a018

[27] Quik M, Choremis J, Komourian J, Lukas RJ, Puchacz E (1996) Similarity between rat brain nicotinic alpha-bungarotoxin receptors and stably expressed alpha-bungarotoxin binding sites. J Neurochem 67:145–154. 8666985 10.1046/j.1471-4159.1996.67010145.x

[28] Smit AB, Syed NI, Schaap D, van Minnen J, Klumperman J, et al (2001) A glia-derived acetylcholine-binding protein that modulates synaptic transmission. Nature 411:261–268. 11357121 10.1038/35077000

[29] Celie PH, Kasheverov IE, Mordvintsev DY, Hogg RC, van Nierop P, et al (2005) Crystal structure of nicotinic acetylcholine receptor homolog AChBP in complex with an alpha-conotoxin PnIA variant. Nat Struct Mol Biol 12:582–588. 15951818 10.1038/nsmb951

[30] Vardjan N, Mattiazzi M, Rowan EG, Križaj I, Petrovič U, et al (2013) Neurotoxic phospholipase A_2_ toxicity model: An insight from mammalian cells. Commun Integr Biol 6:e23600. 23710275 10.4161/cib.23600 PMC3656009

[31] Kasturi S, Rudrammaji LMS, Gowda TV (1990) Antibodies to a phospholipase A_2_ from Vipera russelli selectively neutralize venom neurotoxicity. Immunology 70:175–180. 2115497 PMC1384189

[32] Condrea E, Fletcher JE, Rapuano BE, Yang CC, Rosenberg P (1981) Dissociation of enzymatic activity from lethality and pharmacological properties by carbamylation of lysines in Naja nigricollis and Naja naja atra snake venom phospholipases A_2_ . Toxicon 19:705–720. 6795762 10.1016/0041-0101(81)90108-2

[33] Rouault M, Rash LD, Escoubas P, Boilard E, Bollinger J, et al (2006) Neurotoxicity and other pharmacological activities of the snake venom phospholipase A_2_ OS2: The N-terminal region is more important than enzymatic activity. Biochemistry 45:5800–5816. 16669624 10.1021/bi060217r PMC2796912

[34] Prijatelj P, Pražnikar ZJ, Petan T, Križaj I, Pungerčar J (2008) Mapping the structural determinants of presynaptic neurotoxicity of snake venom phospholipases A_2_ . Toxicon 51:1520–1529. 18513779 10.1016/j.toxicon.2008.03.031

[35] Kini RM, Evans HJ (1989) A model to explain the pharmacological effects of snake venom phosphplopases A_2_ . Toxicon 27:613–635. 2665186 10.1016/0041-0101(89)90013-5

[36] Pungerčar J, Križaj I (2007) Understanding the molecular mechanism underlying the presynaptic toxicity of secreted phospholipases A_2_ . Toxicon 50:871–892. 17905401 10.1016/j.toxicon.2007.07.025

[37] Križaj I, Turk D, Ritonja A, Gubenšek F (1989) Primary structure of ammodytoxin C further reveals the toxic site of ammodytoxin. Biochim Biophys Acta 999:198–202. 2597708 10.1016/0167-4838(89)90218-5

[38] Lambeau G, Schmid-Alliana A, Lazdunski M, Barhanin J (1990) Identification and purification of a very high affinity binding protein for toxic phospholipases A_2_ in skeletal muscle. J Biol Chem 265:9526–9532. 2160984

[39] Lambeau G, Ancian P, Barhanin J, Lazdunski M (1994) Cloning and expression of a membrane receptor for secretory phospholipases A2. J Biol Chem 269:1575–1578. 8294398

[40] Čopič A, Vučemilo N, Gubenšek F, Križaj I (1999) Identification and purification of a novel receptor for secretory phospholipase A_2_ in porcine cerebral cortex. J Biol Chem 274:26315–26320. 10473587 10.1074/jbc.274.37.26315

[41] Cupillard L, Mulhercarn R, Gomez N, Kadam S, Valentin E, et al (1999) Both group IB and group IIA secreted phospholipases A_2_ are natural ligands of the mouse 180-kD M-type receptor. J Biol Chem 274:7043–7051. 10066760 10.1074/jbc.274.11.7043

[42] Augert A, Payré C, de Launoit Y, Gil J, Lambeau G, et al (2009) The M-type receptor PLA2R regulates senescence through the p53 pathway. EMBO Rep 10:271–277. 19197340 10.1038/embor.2008.255 PMC2658567

[43] Bernard D, Vindrieux D (2014) PLA2R1: Expression and function in cancer. Biochim Biophys Acta 1846:40–44. 24667060 10.1016/j.bbcan.2014.03.003

[44] Coenen MJ, Hofstra JM, Debiec H, Stanescu HC, Medlar AJ, et al (2013) Phospholipase A2 receptor (PLA2R1) sequence variants in idiopathic membranous nephropathy. J Am Soc Nephrol 24:677–683. 23431073 10.1681/ASN.2012070730 PMC3609136

[45] Bon C, Changeux JP (1977) Ceruleotoxin: a possible marker of the cholinergic ionophore. Eur J Biochem 74:43–51. 856574 10.1111/j.1432-1033.1977.tb11364.x

[46] Bon C, Changeux JP, Jeng TW, Fraenkel-Conrat H (1979) Postsynaptic effects of crotoxin and of its isolated subunits. Eur J Biochem 99:471–481. 499210 10.1111/j.1432-1033.1979.tb13278.x

[47] Vital Brazil O, Fontana MD, Heluany NF (2000) Nature of the postsynaptic action of crotoxin at guinea-pig diaphragm end-plates. J Nat Toxins 9:33–42. 10701179

[48] Villar MT, Artigues A, Ferragut JA, Gonzalez-Ros JM (1988) Phospholipase A2 hydrolysis of membrane phospholipids causes structural alteration of the nicotinic acetylcholine receptor. Biochim Biophys Acta 938:35–43. 3337815 10.1016/0005-2736(88)90119-8

[49] Ono JK, Salvaterra PM (1981) Snake α-toxin effects on cholinergic and noncholinergic responses of Aplysia californica neurons. J Neurosci 1:259–270. 6114995 10.1523/JNEUROSCI.01-03-00259.1981 PMC6564116

[50] Sine SM, Huang S, Li SX, daCosta CJ, Chen L (2013) Inter-residue coupling contributes to high-affinity subtype-selective binding of α-bungarotoxin to nicotinic receptors. Biochem J 454:311–321. 23802200 10.1042/BJ20130638 PMC3912756

[51] Darveniza P, Morgan-Hughes JA, Thompson EJ (1979) Interaction of di-iodinated 125I-labelled α-bungarotoxin and reversible cholinergic ligands with intact synaptic acetylcholine receptors on isolated skeletal-muscle fibres from the rat. Biochem J 181:545–55752. 518540 10.1042/bj1810545 PMC1161194

[52] Purohit P, Auerbach A (2013) Loop C and the mechanism of acetylcholine receptor-channel gating. J Gen Physiol 141:467–78. 23478996 10.1085/jgp.201210946 PMC3607824

[53] Montecucco C, Gutiérrez JM, Lomonte B (2008) Cellular pathology induced by snake venom phospholipase A2 myotoxins and neurotoxins: common aspects of their mechanisms of action. Cell Mol Life Sci 65:2897–2912. 18563294 10.1007/s00018-008-8113-3 PMC11131735

[54] Kang TS, Georgieva D, Genov N, Murakami MT, Sinha M, et al (2011) Enzymatic toxins from snake venom: structural characterization and mechanism of catalysis. FEBS J 278:4544–4576. 21470368 10.1111/j.1742-4658.2011.08115.x

[55] Mezna M, Ahmad T, Chettibi S, Drainas D, Lawrence AJ (1994) Zinc and barium inhibit the phospholipase A2 from Naja naja atra by different mechanisms. Biochem J 301:503–508. 8042995 10.1042/bj3010503 PMC1137109

[56] Heus F, Vonk F, Otvos RA, Bruyneel B, Smit AB, et al (2013) An efficient analytical platform for on-line microfluidic profiling of neuroactive snake venoms towards nicotinic receptor affinity. Toxicon 61:112–124. 23159399 10.1016/j.toxicon.2012.11.002

